# Improving cost-efficiency of faecal genotyping: New tools for elephant species

**DOI:** 10.1371/journal.pone.0210811

**Published:** 2019-01-30

**Authors:** Stéphanie Bourgeois, Jenny Kaden, Helen Senn, Nils Bunnefeld, Kathryn J. Jeffery, Etienne F. Akomo-Okoue, Rob Ogden, Ross McEwing

**Affiliations:** 1 Agence Nationale des Parcs Nationaux, Libreville, Gabon; 2 WildGenes Laboratory, The Royal Zoological Society of Scotland, RZSS Edinburgh Zoo, Edinburgh, United Kingdom; 3 Biological and Environmental Sciences, Faculty of Natural Sciences, University of Stirling, Stirling, United Kingdom; 4 Institut de Recherche en Écologie Tropicale, Libreville, Gabon; 5 TRACE Wildlife Forensics Network, Edinburgh, United Kingdom; University of Tasmania, AUSTRALIA

## Abstract

Despite the critical need for non-invasive tools to improve monitoring of wildlife populations, especially for endangered and elusive species, faecal genetic sampling has not been adopted as regular practice, largely because of the associated technical challenges and cost. Substantial work needs to be undertaken to refine sample collection and preparation methods in order to improve sample set quality and provide cost-efficient tools that can effectively support wildlife management. In this study, we collected an extensive set of forest elephant (*Loxodonta cyclotis*) faecal samples throughout Gabon, Central Africa, and prepared them for genotyping using 107 single-nucleotide polymorphism assays. We developed a new quantitative polymerase chain reaction (PCR) assay targeting a 130-bp nuclear DNA fragment and demonstrated its suitability for degraded samples in all three elephant species. Using this assay to compare the efficacy of two sampling methods for faecal DNA recovery, we found that sampling the whole surface of a dung pile with a swab stored in a small tube of lysis buffer was a convenient method producing high extraction success and DNA yield. We modelled the influence of faecal quality and storage time on DNA concentration in order to provide recommendations for optimized collection and storage. The maximum storage time to ensure 75% success was two months for samples collected within 24 hours after defecation and extended to four months for samples collected within one hour. Lastly, the real-time quantitative PCR assay allowed us to predict genotyping success and pre-screen DNA samples, thus further increasing the cost-efficiency of our approach. We recommend combining the validation of an efficient sampling method, the build of in-country DNA extraction capacity for reduced storage time and the development of species-specific quantitative PCR assays in order to increase the cost-efficiency of routine non-invasive DNA analyses and expand the use of next-generation markers to non-invasive samples.

## Introduction

Since the early 1990’s, the use of non-invasive DNA analysis has evolved rapidly, allowing the study of species, individuals, gender, kinship and genetic variation [1,2], with clear ethical and practical advantages in endangered or elusive species [3]. With the decrease in laboratory costs per analysis and development of powerful analytical tools, non-invasive genetic population surveys have become increasingly accessible for wildlife management [4–6]. Population censuses based on non-invasive DNA individual identification are more precise and accurate than estimates from indirect signs for a variety of elusive, low-density or wide-ranging species [7–9]. Cost-effectiveness of non-invasive DNA surveys has also been demonstrated [9], but strongly relies on the ability to overcome technical challenges inherent in the use of faecal DNA samples.

The two main technical limitations of faecal sampling are the difficulty of recovering good quality DNA and the high risk of genotyping errors [10–12]. Faecal samples often contain polymerase chain reaction (PCR) inhibitors and low quantities of target DNA, and are prone to DNA degradation and co-recovery of non-target DNA. All of these parameters are strongly influenced by the diet of the sampled individual [13,14] and the environmental conditions affecting the faecal sample in the field. In particular, DNA degrades rapidly in tropical environments due to heat, humidity and a high diversity of microorganisms [15,16].

Attempts to compensate for low DNA extraction success may include increasing the number of faecal samples collected to counteract low success rates [5] and optimizing collection, preservation or extraction protocols [17]. The choice of sampling method and storage conditions (particularly storage media, duration and temperature) strongly influences the quality and quantity of DNA that might be recovered from samples [18]. Numerous sampling and preservation techniques have been extensively tested in a range of species with varying success [18–20], however empirical comparisons have led to a consensus that techniques targeting the outer layer of the dung are generally more efficient [21]. Widely used storage methods include desiccation in silica beads and a variety of liquid storage media, but their efficacy for preserving genomic DNA differs across species and habitat [4]. A two-step protocol consisting of a short period of storage in ethanol followed by silica desiccation has been successfully reported with ungulate and primate samples collected from Central African rainforests [19,22]. In the field of human forensic science, swabs are widely used to collect touched evidence from crime scenes [23] and have proved to be very promising for faecal sampling in a few other taxa and environments [24–26].

Several approaches have been developed to decrease error rates associated with low quality DNA during the amplification process. For example, replicated genotyping (the multiple tube approach) became the gold standard for microsatellite genotyping to minimize allelic dropout in the 1990’s [27], but is costly and incurs significant effort. More recently, single-nucleotide polymorphism (SNP) markers have become widely available [28], with SNP assays less susceptible to genotyping error reducing the need to repeat analysis [29]. Because of this, they are well-suited to non-invasive samples and present a viable alternative to microsatellites [30]. Another approach to balance cost and effort with sample size and error rate is through assessment of faecal DNA samples prior to amplification. The quantification of total DNA alone is not informative enough because faecal samples contain both host and exogeneous DNA, nor is the amplification of one robust marker (e.g. sex marker or 500bp of mitochondrial DNA) sufficient to filter poor quality samples [31,32]. Instead, species-specific quantitative PCR has been developed as a more informative approach to quantify host DNA yield in order to predict the risk of errors and provide critical thresholds for PCR and genotyping replicates [11,23]. In addition, methods to enrich host DNA from faeces have been proposed [33].

Despite these advances in molecular techniques and the variety of tools available, there is little objective evaluation on how to choose between sampling and laboratory methods [17], which precludes the spread of new tools for routine non-invasive genetic analyses. The use of swabs for faecal sampling remains anecdotal among the vast published literature on conservation genetics studies [26], while quantitative PCR assays have been developed in only a limited number of species [34]. To date, relatively few studies have applied SNP genotyping to faecal samples [35–39]. The underuse of these new techniques is one reason why non-invasive genetic approaches arise slowly as routine tools to support conservation management and decision-making [6,40]. Managers are still reluctant to commit resources to faecal DNA surveys because there remains the uncertainty of success in recovering enough good quality data, while a high investment into fieldwork and laboratory costs is required [41]. Substantial work needs to be undertaken to refine sample collection and preparation methods in order to increase the accuracy and success of routine non-invasive DNA surveys and facilitate their implementation for conservation and management.

This paper proposes guidelines to optimize the quality of faecal DNA samples for accurate and cost-effective genotyping. We conducted a non-invasive genetic study using a panel of SNP markers and faecal samples of the endangered forest elephant (*Loxodonta cyclotis*), a relatively understudied species where non-invasive approaches are desirable due to the scarcity of direct observations in a rainforest environment. Our goals were to develop tools for all three elephant species, using an approach that can be applied to multiple taxa, as follows:

1. Development of a quantitative PCR assay;
2. Validation of a new convenient field sampling method with recommendations for sample storage and suitable extraction protocol;
3. Prescreening of a faecal sample set using the quantitative PCR assay and DNA threshold determination for accurate genotyping with a panel of SNP markers.

## Materials and methods

### Sample collection and storage

**Fig 1:**
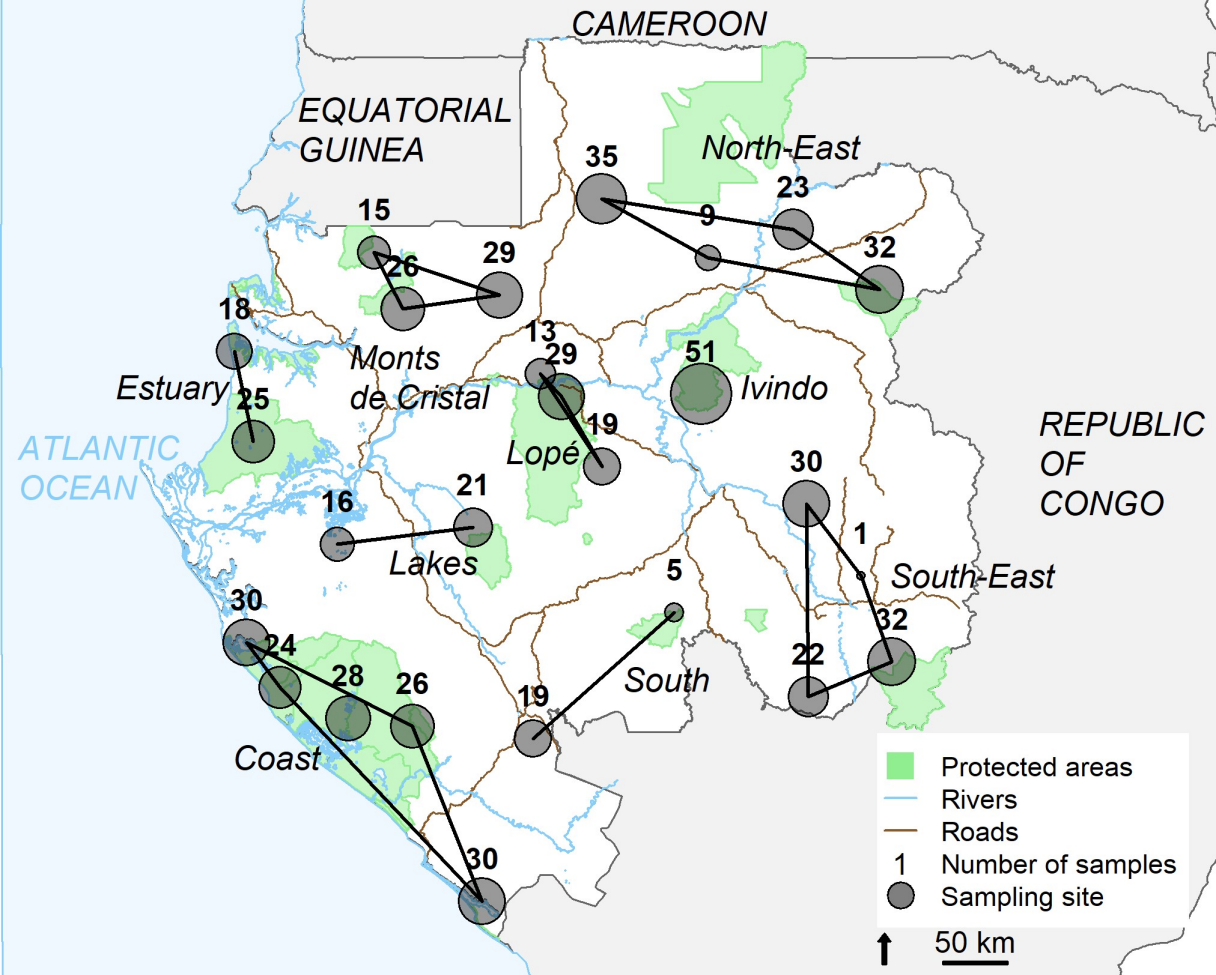
Distribution of elephant sampling locations throughout Gabon. The circles are proportional to the number of faecal samples collected in each sample site (with the total number indicated above). Samples sites were grouped into nine sampling locations (represented by polygons).

We conducted fieldwork between June 2014 and January 2015 at 26 study sites in Gabon, Central Africa (Fig 1). Gabon is mainly covered by tropical forests and 10% of the land has been classified as National Parks. The long rainy season extends from October to April, with a variable short dry season in December and January. The long dry season extends from May to September, although variations occur within the country. Average monthly precipitations ranged from 62 mm to 420 mm. Average monthly temperatures fluctuated between 28°C to 31°C and the mean relative humidity between 88% to 92%. Gabon hosts half of the remaining forest elephant population (~ 50,000 individuals) [42] but faces an unprecedented poaching crisis [43]. The study sites included both National Parks and forestry concessions believed to host high numbers of elephants.

This research was undertaken by the Gabon National Parks Agency (ANPN). We received permissions to conduct this research from the Centre National de la Recherche Scientifique et Technologique (permit AR0016/14) and the Direction Générale de la Faune et des Aires Protégées (certificate of origin 005/15). We obtained access permits from forestry concessions when applicable.

We conducted 1–2 weeks of field surveys within each study site to collect fresh elephant faeces. Faeces were considered “fresh” if they were estimated to be less than 24 hours old, were protected from sunlight by forest cover and had not been exposed to heavy rain. Fresh dung piles were characterized by a shiny colour, mostly intact boli (unless very humid or destroyed by insects) and strong odour [44]. Presence of urine, small flies and elephant footprints in close proximity were other strong indices of freshness. A subset of the fresh faeces was reclassified as “very fresh” (i.e. < one hour old), when the elephant was directly seen or heard and the dung pile was warm.

To evaluate the influence of dung pile quality on DNA extraction efficacy, we also collected samples from faeces that presented a “reduced surface” suitable for sampling (i.e. those classed as less than 24 hours old but partly destroyed by insects or directly exposed to sunlight), and from potentially “degraded” dung piles (i.e. those classed as between 24 and 48 hours old and those of any age that were found after rain or partly immersed in water). For the two latter categories, only the intact shiny surface was swabbed.

**Fig 2:**
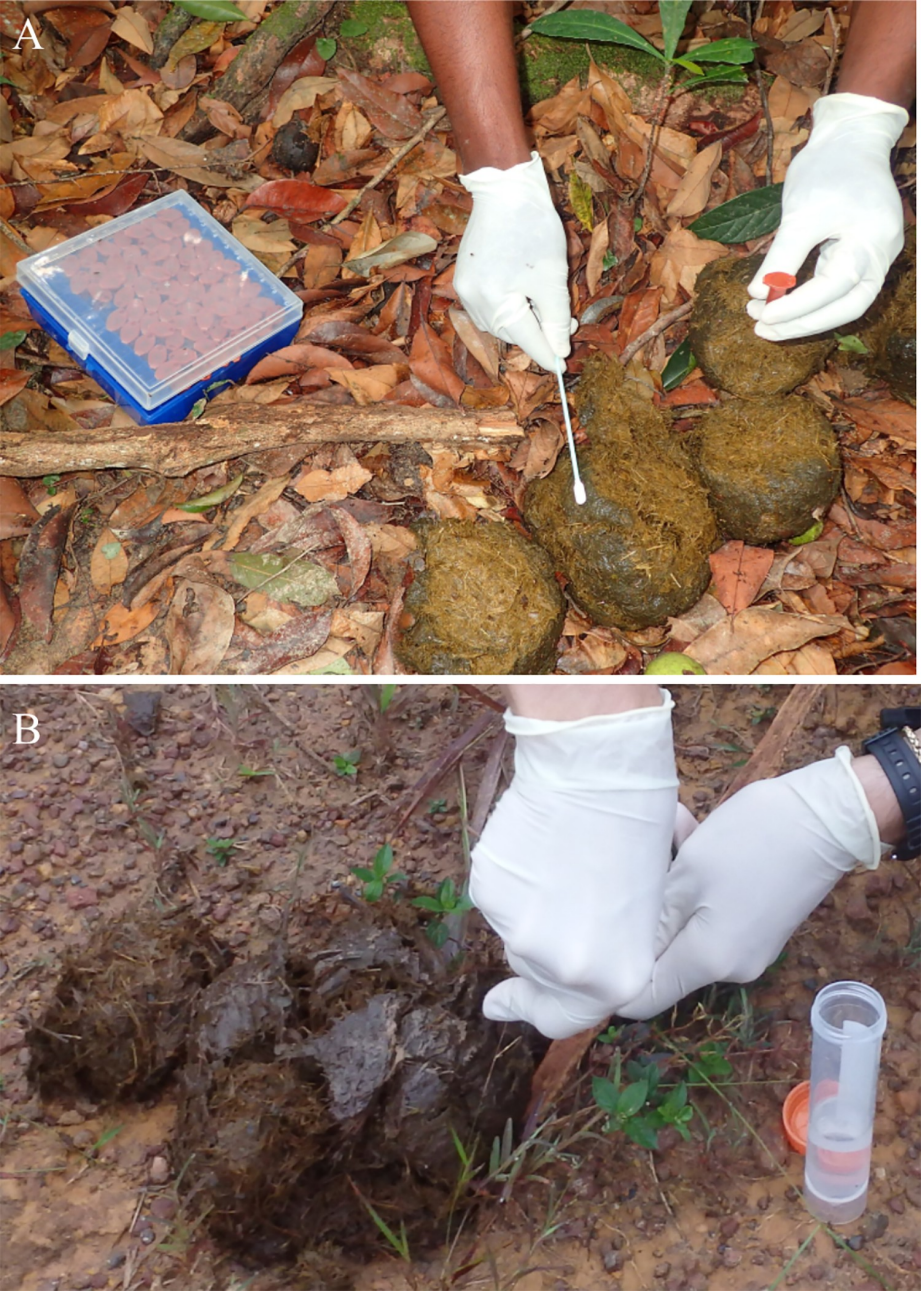
Sample collection from an elephant dung-pile using two sampling methods. Samples were collected using (A) a swab stored in lysis buffer in a 2-ml light protective tube or (B) following a two-step protocol in which a small piece of faeces is stored in ethanol in a 50-ml tube during 24 hours before being transferred into another 50-ml tube with silica beads. The swabbing material was more convenient and easy to carry in the field and allowed to scrub the entire surface of the dung pile.

Faecal samples were collected using a buccal swab (Isohelix, Cell projects) previously moistened with storage buffer (500 μl of LS buffer and 25 μl of proteinase K, Stabilizing Kits, Isohelix, Cell Projects). The entire shiny, mucous surface of every bolus belonging to a dung pile was gently scrubbed with the swab to target the mucous layer coating the dung pile and care was taken to avoid collecting actual faecal material (Fig 2). The swab tip was then snapped and immersed into storage buffer in a labelled 2 ml light-protective Eppendorf safe-lock tube. Samples were stored at ambient temperature in the dark for 1 to 4 weeks before being transferred to the laboratory for immediate DNA extraction or storage at -20°C. As a comparison, we collected duplicate samples from a subset of 78 dung piles using a different sampling method and a two-step preservation protocol (Fig 2). In this method, a small piece of faeces was taken from the outer layer of a bolus and stored in 96% ethanol (20 ml) for 24 hours at ambient temperature before being transferred into silica beads (30 g) [19].

### DNA extraction

We used the QIAamp Fast Stool Mini kit protocol (QIAGEN) to extract DNA from samples preserved using the two-step method, following the manufacturer’s instructions. We modified this protocol to extract DNA from the swabbed samples, as follow: (i) the initial sample (swab tip in buffer solution) was vortexed and centrifuged for 2 minutes (14,100 g) before discarding the swab, (ii) 250 μl of Inhibitex were added to the supernatant, (iii) samples were incubated with proteinase K for 1 hour at 56°C, (iv) 500 μl of CT capture buffer (Isohelix extraction kit, Cell Project) were added to the sample (replacing ethanol), and (v) DNA was eluted in 75 μl of buffer ATE (S1 Table). For every batch of samples, we used DNA extraction blanks to monitor contamination. All DNA extracts were purified using OneStep PCR inhibitors Removal Kits (Zymo research).

### DNA quantification

The concentration of elephant DNA in all samples was measured using a quantitative PCR assay. We designed primers 2804 F (5’CCTGGCAGAGCTCAGCAGAT-3’) and 2804 R (5’GGATGAGGGCCAGAGTGTCC-3’) using Primer3 [45] in Geneious version 9 [46] to amplify a short nuclear sequence (130 bp) of the transmembrane protein 184A gene previously demonstrated to be conserved in forest elephants [47]. We choose the length of the targeted sequence to ensure its suitability for degraded samples and similarity to SNP amplicon size. Faecally-derived DNA samples from two captive African savannah (*Loxodonta africana*) and five captive Asian (*Elephas maximus*) elephants were included in the analysis to test for efficiency of the primers in these species. We used BLAST (Basic Local Alignment Search Tool) to confirm that the primers did not amplify human DNA.

Seven serial dilutions of DNA extracted from a forest elephant tissue sample provided standards to calibrate absolute quantification. The serial dilution ranged from 20 to 0.0013 ng/μl with a serial factor of 5. The four highest standards (20, 4, 0.8 and 0.16 ng/μl) were stored at 7°C for 48 hours to ensure homogenisation and quantified by fluorometry (using QuBIT DNA Broad Range and High Sensitivity Assay kits, Invitrogen, Thermo Fisher). The three lowest standards were freshly prepared before the experiment by serial dilution and vortexed to ensure homogenisation before the subsequent dilution. Standards and negative controls were included in duplicate in all plates. All quantitative PCR experiments were performed over a period of four days in order to minimize the variation of standards between plates and two positive controls were repeated across plates to check for variability. A subset of faecal samples were re-run in pairs of swabbed samples with duplicated two-step preserved samples over a two-day period with fresh standards. In addition, we quantified a subset of 27 samples by fluorometry (using QuBIT DNA Broad Range and High Sensitivity Assay kits, Invitrogen, Thermo Fisher) in order to compare total and elephant DNA yield.

Quantitative real-time PCR reactions were conducted in 10 μl reactions containing 1 μl of DNA, 5 μl of SYBR Green I Master mix, 1 μl of QN ROX Reference Dye (QuantiNova SYBR Green PCR Kit, Qiagen) and 0.7 μl of each primer (10 μM). To dilute inhibitors [38], faecal samples were diluted 1 in 20 with double distilled water before the experiment. Quantitative PCR reactions were carried out on a StepOne Real-time PCR system (Applied Biosystems) with an initial holding step of 2 min at 95°C, followed by 40 cycles of 95°C for 5 s, 60°C for 10 s and a final melt curve stage gradually increasing from 60 to 95°C for 15 minutes. Standard curves were used to calculate elephant DNA concentration in the 20 x diluted samples [11]. The converted concentration of the neat DNA extracts was used for further analyses, unless otherwise stated. Efficiency of the standard curves (correlation coefficient r^2^) and melt curve profiles were examined. Any standard or sample generating non-specific amplification (i.e. PCR products that melt at temperatures above or below the desired product 84.7°C) were discarded from the analysis.

### Genotyping

In order to assess genotyping success, samples were sent to LGC Genomics for SNP genotyping using 107 KASP assays developed and validated for forest elephants [47]. A pilot study was performed using four SNP assays (CL_370, CL_406, CL_2831 and CL_2968) and several dilutions (5, 10, 20, 40) of a subset of 88 samples selected over a wide range of concentrations (0 to 12.2 ng/μl). In order to determine the optimal dilution, we classified the samples into four categories based on target DNA concentration: [0–0.01), [0.01–0.1), [0.1–0.6) and ≥ 0.6 ng/μl. We estimated the mean genotyping success at four loci at each dilution factor for all categories. Based on this preliminary testing, further genotyping was performed using 10 x dilutions of all faecal samples and all samples that yielded a concentration above 0.01 ng/μl were selected for genotyping (S1 Fig). To test if elephant DNA concentration predicted genotyping success, a random subset of samples with very low DNA yield (0–0.01 ng/μl) were also selected for genotyping. Genotype scoring was conducted by automatic allele calling (LGC Genomics). In order to control for quality, two negative controls were included in each 96-well plate and 14 samples were replicated two or three times in different plates. We assessed the allelic error rate directly as the proportion of allelic dropout and false alleles within the positive controls.

### Data analyses

We estimated the extraction success as the proportion of samples with a detectable elephant DNA yield using the quantitative PCR assay and the genotyping success per sample as the proportion of loci for which an unambiguous genotype was assigned. Using the subset of 78 duplicate dung samples, we compared elephant DNA concentrations from samples collected by the swab and two-step protocols using a nonparametric Mann-Whitney-Wilcoxon test. We also evaluated statistical differences across the two sampling methods within the different faecal quality groups.

We used generalized linear mixed models to test the influence of storage time and faecal quality as independent predictor variables on elephant DNA concentration. As the frequency plot suggested zero-inflation (S2 Fig), we used a two-part model in order to investigate the influence of storage time and quality on both DNA presence and concentration [48]. In the first part, we used a binomial distribution to model the probability that a zero value is observed and we used the model to predict extraction success against storage time for different DNA qualities. In the second part, we fitted a truncated negative binomial distribution to the non-zero data to account for over-dispersion and we used the model to test the influence of storage time and faecal quality on elephant DNA concentration.

The response variable was the absolute value of elephant DNA concentration in pg/μl. Quality types included “very fresh”, “fresh”, “reduced surface” and “degraded” faeces. Fresh quality was used as the reference category. We used storage time (in weeks) as a continuous variable (standardized). We also included an interaction between storage time and quality in the model to test if the influence of storage time varied with faecal quality. Storage time was highly correlated with season due to logistical constraints so we excluded the latter from the model. Study sites were grouped into nine locations when they were close (Fig 1) and visited at the same season. All samples from one location were collected, transported to the laboratory and extracted simultaneously as a batch. Therefore, to correct for the lack of independence between samples collected within the same location and account for other possible effects (e.g. weather, diet, habitat type, transport conditions), we treated sampling location as a random effect. We used the Akaike Information Criterion (AIC) to compare candidate models and choose the minimal adequate model [49].

We used quasi-binomial generalized linear models to examine the influence of target DNA concentration on genotyping success for different panels of 15, 50 and all 107 SNPs and determine concentration thresholds for genotyping. DNA concentrations were log transformed for statistical analyses. Panels of 15 and 50 SNPs were selected based on highest genotyping success per locus. All analyses were conducted using R version 3.3.1 [50], using the packages lme4 [51] and glmmADMB [52,53].

## Results

In total, 572 faecal samples, including 458 fresh dung samples were collected using the swabbing technique. Median storage time between sample collection and DNA extraction was 7.6 weeks (range: 0.7–18.9). Following quantitative PCR, all standard curves showed good accuracy (r^2^ > 0.95). All three elephant species amplified successfully using the 2804 primers demonstrating the conserved nature of this fragment. Faecal DNA concentrations for fresh swab samples ranged from 0.0 to 26.99 ng/μl (mean = 0.97 ng/μl, n = 458). The proportion of endogenous to total DNA ranged from 0.001 to 29.5% (mean = 2.93%, n = 27). The overall extraction success for fresh samples was 65.9% (n = 458). It rose to 74.5% (n = 47) for very fresh samples collected within one hour of defecation and 84.7% (n = 261) for fresh samples extracted within 8 weeks. Following DNA extraction, the colour of 76 DNA eluates was brown and failure of quantitative PCR reactions indicated the presence of inhibitors. These samples were excluded from further analyses. In total, 382 samples yielded a target elephant DNA concentration above 0.01 ng/μl and were genotyped at all SNP loci, along with 121 samples that didn’t reach this threshold. Following genotyping at 107 loci, the error rate was 0.0029.

### Comparison of sampling methods

**Fig 3:**
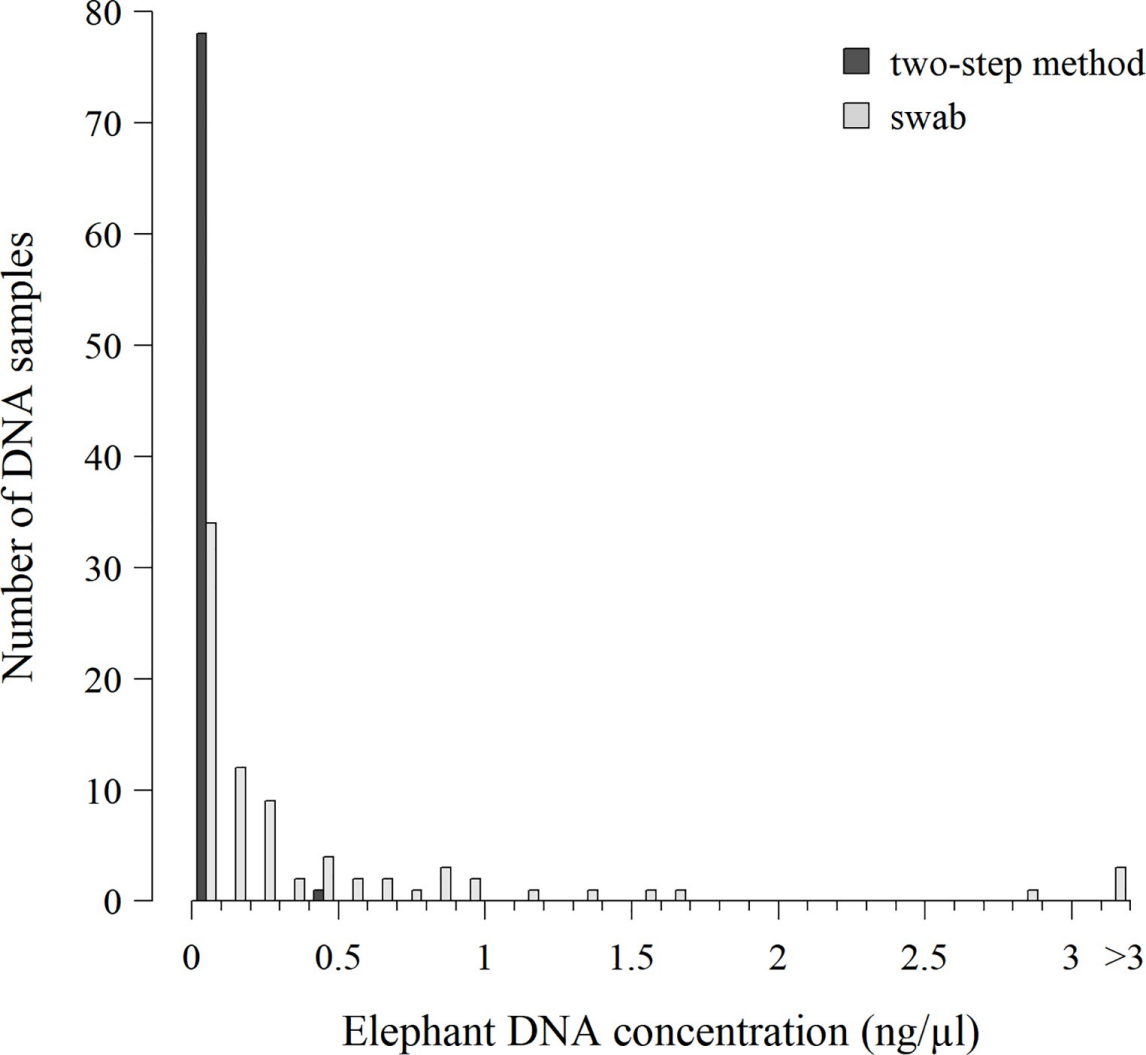
Distribution of elephant DNA concentration in 79 faecal samples collected in duplicates using a swab or a two-step method. Elution volume was 75 μl for all samples.

The elephant DNA concentration in swabbed samples was 42.9 times higher than in silica-preserved samples and the difference was statistically significant (V = 1631, p < 0.001) (Table 1). Higher target DNA concentration was also obtained with the swabbing technique in all categories of faecal quality (p < 0.05). Median concentration was between 29.5 (< 1 hour) and 505.4 (> 24 hours) times higher in swabbed samples than in silica gel-preserved samples. Maximum elephant DNA concentration obtained from samples preserved using the two-step method was as low as 0.47 ng/μl and only 5 samples reached the DNA concentration threshold of 0.01 ng/μl (Fig 3).

**Table 1:** Results of the Mann-Whitney-Wilcoxon test on mean elephant DNA concentration (ng/μl) between two faecal DNA sampling methods. The results are based on 79 faecal samples collected in duplicates using a swab or a two-step protocol. Samples were classified into four categories based on faecal quality: very fresh, fresh, reduced surface and degraded.

Quality	Mean two-step	Mean swab	V	p-value
All (n = 79)	0.011±0.544	0.471±1.032	1631	<0.001
Very fresh (n = 10)	0.056±0.145	1.668±2.286	45	0.009
Fresh (n = 33)	0.007±0.021	0.478±0.713	290	<0.001
Reduced surface (n = 22)	0.004±0.011	0.165±0.226	150	<0.001
Degraded (n = 14)	0.000±0.000	0.095±0.102	28	0.022

### Influence of storage time on target DNA concentration

**Fig 4:**
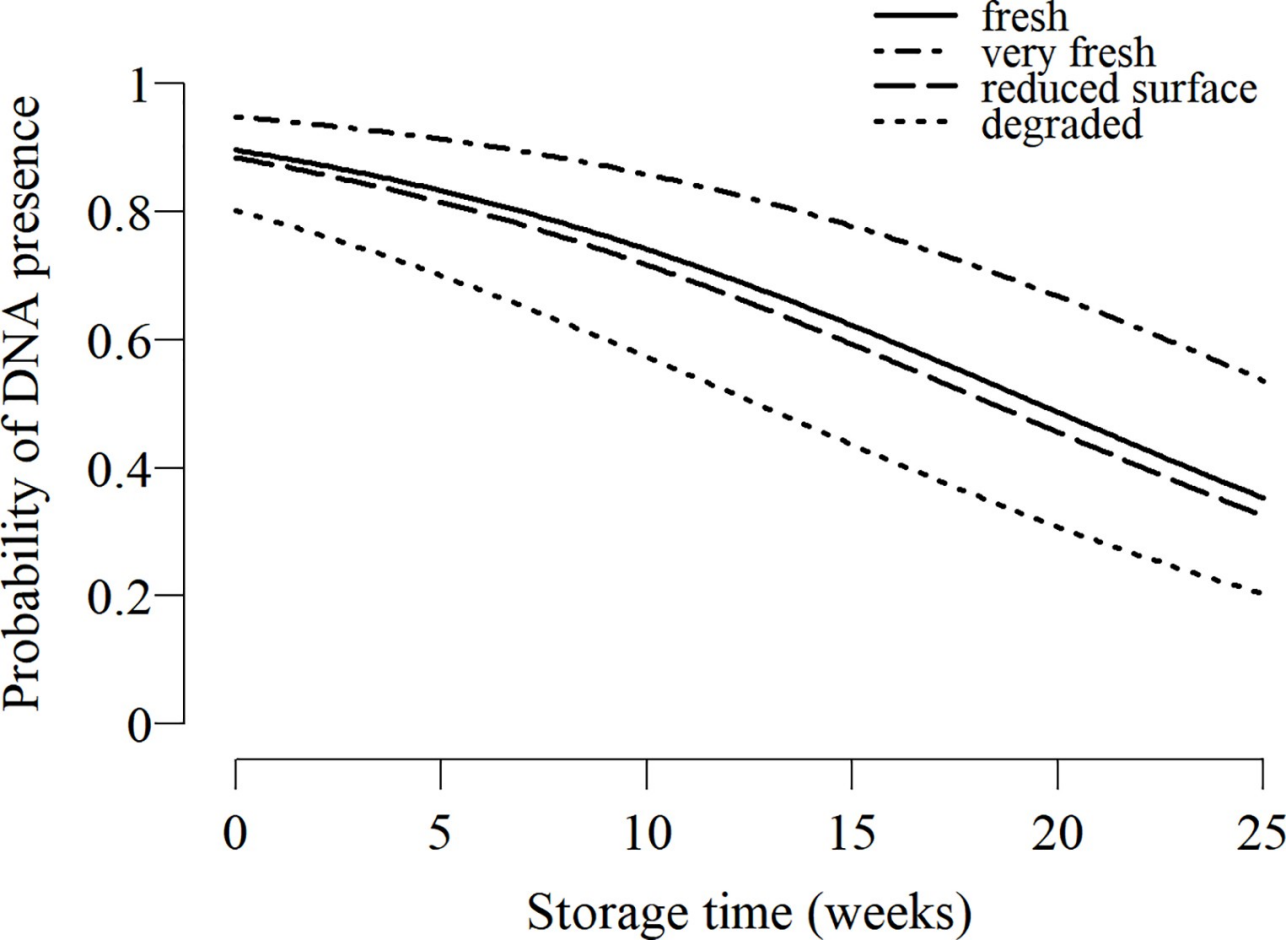
Predicted probability to extract elephant DNA from faeces per week of storage for different faecal qualities. Faecal quality categories included: very fresh (collected within 1 hour after defecation), fresh (collected within 24 hours after defecation), reduced surface (less than 24 hours old but partly destroyed by insects or directly exposed to sunlight), and degraded (collected between 24 and 48 hours after defecation or found after rain or partly immersed in water). Details of the model are given in Table 3.

In the binomial model explaining DNA presence, the two best models based on AIC included only storage time or both storage time and quality effects (ΔAIC<2). The interaction term did not significantly improve the model (ΔAIC = -2.1) (Table 2). We used the model with storage time and quality to model DNA presence because it had the lowest AIC and the “degraded” category was significantly different (p < 0.05) (Table 3). Storage time of the faecal sample had a significant influence on the probability of DNA presence in the extract (p < 0.001) (Table 3). Degraded dung piles were 2.13 times less likely to provide DNA than fresh dung piles (p < 0.05). The difference between very fresh, fresh and reduced surface faeces was not significant. The random effects explained 14.1% of the variance. Extraction success was 11.3% and 12.3% lower in samples collected in two of the locations (South and Coast) (S3 Fig). The model predicted that the extraction success declined to 75% after 9.5 weeks of storage. The predicted success dropped to 50% after 19.5 weeks of storage for samples collected from fresh faeces, against 12.6 weeks from degraded faeces and > 6 months from very fresh faeces (Fig 4). The prediction fitted well to observed data with the exception of a batch of 47 samples from the Coast, for which success was only 31.9% (S4 Fig).

**Table 2:** Comparison of candidate binomial models for the prediction of elephant DNA presence in faecal samples. Variables are storage time (t) (standardized) and faecal quality categorized into four groups: very fresh (Qvf), fresh (reference category), reduced surface (Qs), degraded (Qd).

Model	Intercept	t	Qvf	Qs	Qd	t*Qvf	t*Qs	t*Qd	AIC
Intercept-only	1.34*								521.4
Storage time	1.27*	-0.62*							510.3
Faecal quality	1.40*		0.58	-0.30	-0.71*				521.2
Storage time +faecal quality	1.29*	-0.65*	0.75	-0.12	-0.78*				508.7
Storage time *faecal quality	1.29*	-0.72*	1.36	-0.19	-0.75*	-0.77	0.32	0.35	510.8

AIC, Akaike information criterion.

*Parameter values of candidate models are marked by an asterisk if significant at the 5% level.

**Table 3:** Summary of the best binomial generalized linear mixed model for the effects of storage time and faecal quality on elephant DNA extraction success. Faecal quality of 496 faecal DNA extracts was categorized into four groups: very fresh, fresh (reference category), reduced surface and degraded. Sampling location was included as random effect.

Variable	Coeff. (±SE)	Z	p-value
Fixed effects			
Intercept	1.288 ±0.210	6.133	<0.001
Storage time	-0.653 ±0.156	-4.179	<0.001
Very fresh	0.751 ±0.502	1.496	0.135
Reduced surface	-0.124 ±0.376	-0.331	0.741
Degraded	-0.757 ±0.362	-2.088	0.034
Random effects			
No. groups	9		
Variance	0.164		
SD	0.405		

In the model containing data above zero, the model with the lowest AIC indicated that elephant DNA concentration was influenced by storage time, faecal quality and an interaction effect between the two variables (Table 4). The results were less strong than the binomial model due to small sample size in three quality categories and noise, but confirmed similar patterns to the first part of the model (results presented in S1 Appendix).

**Table 4:** Comparison of candidate truncated negative binomial models for the prediction of elephant DNA concentration in faecal samples. Variables are storage time (t) (standardized) and faecal quality categorized into four groups: very fresh (Qvf), fresh (reference category), reduced surface (Qs), degraded (Qd).

Model	Intercept	t	Qvf	Qs	Qd	t*Qvf	t*Qs	t*Qd	AIC
Intercept-only	6.52*								6339
Storage time	6.47*	-0.28							6327
Faecal quality	6.43*		1.02*	-0.59*	0.05				6251
Storage time +faecal quality	6.36*	-0.30*	1.12*	-0.53*	0.02				6234
Storage time *faecal quality	6.43*	-0.13	0.96*	-0.63*	-0.18	-0.44*	-0.23	-0.56*	6217

AIC, Akaike information criterion.

*Parameter values of candidate models are marked by an asterisk if significant at the 5% level.

### Influence of DNA concentration on genotyping success

**Fig 5:**
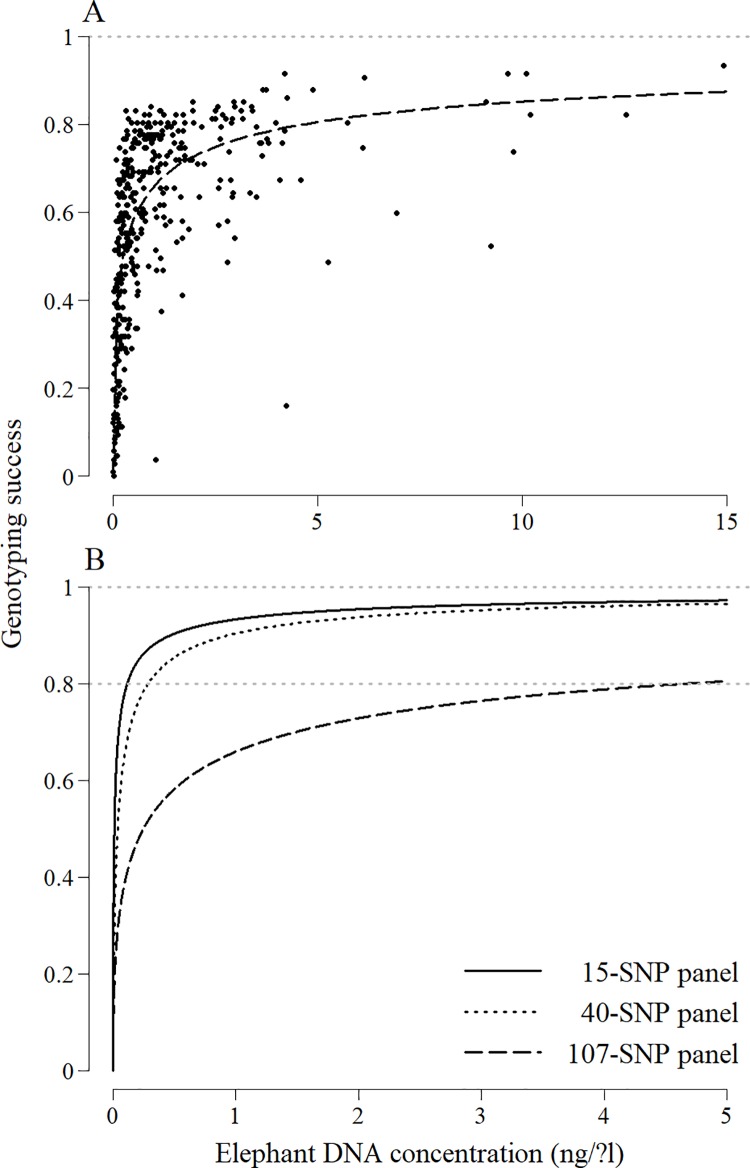
Relationship between the genotyping success using different SNP panels and elephant DNA concentration. (A) The relationship between the genotyping success at 107 SNP loci and elephant DNA concentration measured using a real-time quantitative PCR assay was established using a dataset of 521 faecal DNA extracts (represented by points). (B) This relationship was compared to smaller panels of 15 and 40 SNPs. Genotyping was performed for each locus using 1.5 μl of a 1:10 dilution of DNA extracts.

Genotyping success was significantly correlated with elephant DNA concentration (p < 0.001) (Fig 5A). The model predicted that a concentration of 4.65 ng/μl (698 ng per reaction) resulted in a 80% genotyping success with the panel of all 107 SNPs. Target concentration thresholds were lower for smaller SNP panels (Fig 5B). Minimum concentrations of 0.285 and 0.115 ng/μl (42.8 and 17.3 pg DNA per reaction) were required to reach a genotyping success of 80% with a panel of 40 and 15 SNPs, respectively. Our threshold of 0.010 ng/μl (1.5 pg per reaction) resulted in a 49.3% genotyping success with a panel of 15 SNPs.

## Discussion

Despite the need for non-invasive tools to monitor wildlife populations, faecal genetic sampling is not routinely used as a wildlife management tool, largely because of the associated technical challenges and cost. Optimization work is required at all steps from sample collection to DNA preparation for genotyping in order to improve cost-efficiency and dataset quality. In this study, we collected an extensive set of forest elephant faecal samples and assessed their suitability for genetic analyses. Through a newly developed quantification assay, we demonstrated the efficiency of new sampling and extraction protocols in elephants. As expected, the real-time quantitative PCR assay allowed us to predict genotyping success and pre-screen DNA samples.

### Optimizing field sampling protocols

Choice of sampling technique and storage medium are crucial for subsequent genotyping success. Our results show that swabbing the dung surface followed by storage into a lysis buffer was an effective sampling technique, consistent with previous studies of other species [24,54,55]. Despite these promising results and their convenience in the field, swabs have been relatively little-used in faecal genetic sampling of wildlife [23,26]. To our knowledge, this study is the first to report the use of swabs for faecal sampling in elephants and the observed DNA extraction success for samples collected within 24 hours of defecation was high (85% within 8 weeks of storage). This is higher than reported in other studies of forest elephants, where 60 to 80% of faecal samples stored in ethanol or in Queen’s college buffer, which is recommended by the CITES MIKE (Monitoring the Illegal Killing of Elephants) programme [56], were successfully used for microsatellite genotyping [9,44,57–59].

We found that target DNA yield was more than 40 times higher in swab samples compared to samples preserved using a two-step method, irrespective of faecal quality. Based on elephant DNA concentration, we would therefore have discarded 79.5% of the samples collected using the two-step method before genotyping. Only two other studies have made a direct comparison between swabbing and other sampling techniques. Similar to our results, in equids, genotyping success was nearly zero with the two-step method and almost 100% with swabs [17]. Higher target DNA yield has been reported with swabs compared to ethanol storage in several species, especially in herbivores [26].

The high efficacy of the swabbing method demonstrated in our study might be explained by the sample collection technique. We used the swab to scrub the entire surface of the dung, thus yielding more DNA per sample than techniques targeting a small piece of the outer layer of the dung. This is especially true in species with large scats or numerous pellets providing a greater surface area [60]. In addition, our findings illustrated the efficiency of the swabbing technique to target host cells, as the proportion of endogenous DNA was high compared to values reported in other studies using faecal samples collected with other techniques [26,61]. Swabs target sloughed intestinal epithelial cells at the surface of the dung more specifically than other collection techniques, thus reducing the simultaneous collection of diet or microbial material [54]. The relatively low proportion of endogenous DNA in our study (up to 29.5%) compared to values around 50% reported in swab samples in another study [26] might be limited by a higher concentration of microorganisms at the surface of the dung in a tropical environment.

The choice of dung piles that are suitable for sampling is another crucial step determining the success of sample collection. We found that the extraction success of swab samples was influenced by dung pile freshness and exposure to various environmental factors, which included UV light, humidity, as well as unmeasured factors such as temperature and microorganisms, as expected from previous studies [13,16,62,63]. We showed that freshness had a major impact on elephant DNA concentration and, therefore, sample quality for DNA studies was optimal within one hour after defecation. This was contrary to previous findings in otter (*Lutra lutra*), where no variation was detected within 20 hours after defecation [54], but likely due to the tropical environment, as degradation happens quicker than in dry or very cold environments [13]. In our study, the extraction success declined due to the humid environment (rainfall or partial immersion into water) and the age of dung sample exceeding 24 hours, which was also reported in tigers (*Panthera tigris*) [64]. In contrast, exposure to direct sunlight significantly reduced DNA concentration but not DNA presence, and these samples were suitable for genotyping. This outcome might be explained by our sampling technique, as we swabbed only the sides of the dung that were shaded from direct UV light.

In our study, differences among locations and individuals also explained a part of the variability in both models of DNA presence and concentration. These differences could be explained by variations in diet, which is known as a factor influencing genotyping success [14]. Previous studies suggested that diet quality influences the digestion time, and thus the abrasion of intestinal cells that contain host DNA [65], and that some plants or fruits contain PCR inhibitors [66,67]. In our study, fruit species and the proportion of grass in elephant faeces varied among sampling sites and seasons (S. Bourgeois, personal observation). More research is needed to help select dung piles that are most suitable for DNA studies based on elephant diet.

A major outcome of our study is the reduction in cost and effort for generating a high quality faecal genetic dataset. The swabbing material was more convenient and easy to carry, requiring minimal space in the field, thus allowing to collect more samples in one field trip, representing a 50% reduction in field man-days in remote areas. These are strong advantages for remote and difficult to access field sites, such as tropical rainforests. In addition, the high extraction success reduces the targeted number of dung samples usually necessary to compensate for analytical failure [5], which further decreases field costs and effort by about 15%. In species with low density and/or daily defecation rate, the reduction in field costs might be limited by the difficulty to find fresh dung samples (< 24 hours). In addition, the age of dung piles might be difficult to evaluate in the field [68]. In these species, it may be necessary to collect older dung samples in order to increase the number of samples collected, even though this leads to an increase of laboratory costs due to a decreased extraction rate. A pilot study including dung samples of various ages would allow to set reasonable thresholds for dung age in these species, as a balance between laboratory costs and field efforts.

In the laboratory, DNA extraction from swab samples was fast and straightforward, as the tube was simply vortexed for 10 seconds and centrifuged for 2 minutes before the swab was discarded. In contrast, DNA extraction from samples collected using the two-step protocol was time consuming and involved a higher risk of contamination, due to the need to scrape or choose a piece of faeces prior to the extraction. The number of swab samples that could be extracted per day per person was 48 with the swab samples, compared to only 16 with the two-step protocol, representing a 66% reduction in labor costs. This was similar to results from a previous study showing that DNA extraction from swabs was associated with faster processing times and allowed to work with larger batch sizes [69].

The marked advantages of sampling fresh faecal material in terms of laboratory success should also be considered in relation to the increased effort in finding sufficient samples of this type, as opposed to more relaxed criteria for collecting faecal material in any condition. There is always a trade-off in terms of project cost between sample collection and laboratory analysis. Laboratory analysis is easier, quicker and cheaper when using reliable DNA sample sources, but while this results in a preference for invasive samples types over non-invasive samples, and fresh non-invasive faecal material over older material, lab efficiencies due to high sample quality may be offset by elevated field costs. However, the trade-off has some hard borders. Just as it is considered completely impractical (financially and ethically) to tranquilize wild forest elephants to get the best possible quality of DNA sample, it is simply not possible to perform DNA analysis on samples in which the DNA is completely degraded. As this point is approached, the cost of DNA analysis increases, but also, importantly, the quality of the resulting genetic data and its utility in biological inference decrease. This issue of data quality is often overlooked in a simple cost trade-off between lab and field expenses. We would therefore argue that higher search effort in the field to find fresh samples is actually a requirement, rather than a balanced choice, if the alternative is the collection of samples which are not only very expensive to process in the lab, but also only yield data of marginal biological value. It is important that this issue is widely understood to improve fieldwork planning and to manage expectations of wildlife managers and donors when embarking on conservation genetic projects.

### Optimizing sample preparation

We highlighted the importance of sample preparation, including faecal sample storage before DNA extraction and DNA sample dilution prior to genotyping, by investigating the effects of storage time and dilution rate on genotyping success. We showed that storage time negatively influenced DNA extraction success and we used this relationship to provide recommendations for maximum storage time. Elephant DNA concentration also decreased with increased storage time even if there was high variability among samples. Predicted faecal DNA extraction success declined to below 75% after two months and 50% after five months. This finding was similar to other studies that show a significant reduction in genotyping success after one to three months of storage irrespective of storage medium [20,70]. PCR success rates of 75% were obtained with DNA extracts stored for up to four months, and 50% for those stored for more than six months, when dung piles were sampled within 1 hour of deposition. This highlights the importance of selecting the freshest dung possible, although admittedly this is not always practical for elusive species. Some authors have suggested removing the cotton swab for long-term storage [55]. Storage of samples at lower temperature, such as -80°C, might also slow DNA degradation. However, we believe that a short storage time is a key factor in the success of genetic surveys.

Careful planning for laboratory analyses prior to conducting fieldwork is paramount in order to limit storage time and increase DNA extraction success. Building in-country capacity for DNA extraction in a source country would allow to process samples as they are collected, which is especially important in studies involving a long fieldwork period where regular export of samples is impractical. The required investment in basic equipment and training is reasonable. A DNA extraction laboratory may be set up in one room equipped with a bench, a set of pipettes, a centrifuge, an incubator, a vortexer and a freezer (total cost < 6,000 USD) and ready-to-use DNA extraction kits. Training of a lab technician in DNA extractions may be possible within a couple of weeks.

Beside a low extraction success leading to absence or insufficient target DNA yield, the presence of inhibitors is the second most common cause of amplification failure in faecal samples, under validated PCR conditions [3]. Our study highlighted the need to conduct a pilot study to determine the optimal dilution prior to genotyping. The pilot study showed that a 10x dilution increased the genotyping success, which was similar to a previous study in Asian elephants [38]. The optimal dilution was a compromise between the appropriate dilution of inhibitors in samples with a high DNA yield while minimizing the risk of diluting DNA in samples with a low DNA yield. However, in our study, a substantial subset of DNA eluates (14.5%) exhibited a brown colour, which is often associated with the presence of inhibitors such as humic contaminants [71]. These samples could not be quantified using the PCR assay at any dilution rate. As the provenance of these samples were concentrated in 5 sites (2 sites in the Estuary, 1 site along the Coast, Lopé and Lakes), we believe this was due to variations in diet and not to the sampling method. The swabbing technique was rather found to minimize PCR inhibitors [26]. Future research should be directed to improve extraction protocols, in particular purification steps in order to optimize the removal of inhibitors [72].

### Prescreening DNA samples prior to genotyping

We found that despite optimized sample collection, preservation and extraction protocols, the quality and quantity of DNA extracted from dung piles varied greatly across samples. Therefore, a prior assessment of samples was needed to increase the overall genotyping success and decrease the risk of errors. Target DNA yield was a good predictor of genotyping success, as shown in previous studies [11,22,73,74]. When the species of origin is difficult to confirm by visual examination of the dung (e.g. in carnivores), prior identification of the species is required and often involves mitochondrial DNA sequencing [16,60]. A two-step approach starting with mitochondrial DNA sequencing to inform the subsequent choice of an appropriate species-specific quantitative PCR assay, may be a cost-effective technique for prescreening the samples based on concentration. This would require thorough testing of primer specificity to ensure they do not amplify DNA from related species [75,76]. Alternatively, a single-step option would be to differentiate among multiple species (e.g. carnivores) by combining carnivore-generic PCR primers with species discriminatory melt-curve analysis in a single qPCR assay.

By simulating two different reduced panels of 15 and 40 SNPs using high quality loci and numbers of loci commonly used for individual identification or parentage analyses [77,78], we showed that the relationship between target DNA concentration and genotyping success varied across number of markers and individual loci. Our approach was conservative, as we didn’t rescore the genotype plot manually. This would have increased the genotyping success, because automatic allele calling results in a high proportion of unassigned genotype calls [79]. Despite this, we found that very low amounts of DNA per reaction was required to achieve 80% genotyping success (22.5 or 45 pg DNA per reaction with a panel of 15 or 40 SNPs, respectively). These values were lower than cut-offs reported for microsatellite and SNP genotyping (50–200 pg per reaction) in previous studies [11,22,60,80]. Differences in thresholds between studies are explained by variation in the type of markers [73], choice of genotyping assay [35] and species of interest [74]. As a consequence, thresholds for sample categorization need to be established on a case-by-case basis for each species and set of markers. Our study re-emphasized the need to conduct a pilot study [10,34] in order to set reasonable thresholds. A pilot study would also allow estimating the proportion of bad quality samples and decide if they should be discarded or genotyped in replicates, as a balance between costs and the need of a suitable sample size.

The use of quantitative PCR has long been limited by equipment and reagent costs [34] but this technique is now affordable [23]. Discarding samples unlikely to produce viable results before genotyping reduces the genotyping costs and the risk of errors. DNA quantification (reagents and plates) costs approximately US$ 1 per sample, excluding labour costs, and allowed us to reduce overall genotyping costs by more than a third. The need for a prescreening of samples is even higher in species where the age of dung piles is difficult to evaluate in the field [68], thus leading to a higher proportion of unsuitable samples. In other studies, up to 50–60% of non-invasive samples have been discarded based on target DNA quantification [74,81,82]. The cost reduction is even greater when compared to the multi-tube approach advocated for microsatellite studies [10], where the recommended 7 replications for homozygous loci is often prohibitively expensive. For example, a quantitative PCR assay was used to reduce the number of replications required for accurate genotyping down to 2 for samples above DNA quantity thresholds [11]. We believe that the development and use of species-specific quantification assays would strongly increase the cost-efficiency of faecal DNA surveys.

## Conclusions

We demonstrated the efficiency of our tools in generating a good quality faecal DNA dataset in elephants. Therefore, we recommend the collection of faecal DNA samples within 24 hours of defecation for elephant species using a swab preserved in lysis buffer. DNA extraction should be performed as soon as possible after collection or within two months to ensure 75% extraction success. The use of the quantitative PCR assay, that was validated in all three elephant species, to pre-screen the DNA samples is valuable to reduce the cost of genotyping.

The same approach might be used by managers to improve the cost-efficiency of routine faecal DNA surveys in a wide variety of species. In order to optimize the quality of faecal DNA samples from the field to the laboratory for accurate and cost-effective genotyping, we recommend to:

1. Validate an efficient and convenient sampling technique in the species and environment of interest. We strongly recommend testing the swabbing technique and expect that its use will rise in future studies of elephants and other species;
2. Perform DNA extraction as soon as possible after sample collection to ensure suitable DNA yield. In many cases, the development of an in-country capacity for DNA extraction would be instrumental in reducing storage time;
3. Conduct a pilot study to assess optimal dilution to minimize the effects of inhibitors and determine a threshold for successful and accurate genotyping using a chosen set of markers;
4. Quantify target DNA in all samples and discard poor quality samples before genotyping.

We believe this approach will help managers widely embrace faecal DNA surveys and contribute to a shift towards the field of genomics using faecal DNA.

## Supporting information

S1 Table A modified protocol for DNA extraction from faecal samples collected using a swab. Faeces were scrubbed using a buccal swab (Isohelix, Cell projects) and preserved into storage buffer (500 μl of LS buffer and 25 μl of proteinase K, Stabilizing kit, Isohelix, Cell Projects). The protocol is derived from the QIAamp Fast Stool Mini kit (51604) protocol (QIAGEN) and the Isohelix DNA Isolation kit (DDK-50) protocol (Cell Project).
(DOCX)

S2 Table Sampling and laboratory data. (CSV)

S1 Fig Genotyping success at 4 SNP loci at four dilutions (1:5, 1:10, 1:20 and 1:40). The pilot study included 88 faecal DNA extracts DNA extracts classified into four categories based on target DNA concentration.
(TIFF)

S2 Fig Distribution of elephant DNA concentration in 496 fecal samples collected using a swab. DNA concentrations ranged between 0 and 28.0 ng/μl. Samples were preserved into a lysis buffer and elution volume was 75 μl for all samples.
(TIFF)

S3 Fig Coefficients of random effects for the 9 sampling locations in the two-part model. The model included (A) the best binomial generalized linear mixed model for the effects of storage time and faeces quality on elephant DNA extraction success, and (B) the best truncated negative binomial generalized linear mixed model for the effects of storage time and faeces quality on elephant DNA concentration.
(TIFF)

S4 Fig Observed and predicted probability to extract elephant DNA from faeces per week of storage for different faecal qualities. The four categories of faecal quality were: (A) very fresh (collected within 1 hour after defecation), (B) fresh (collected within 24 hours after defecation), (C) reduced surface (less than 24 hours old but partly destroyed by insects or exposed to direct sunlight), and (D) degraded (collected between 24 and 48 hours after defecation or found after rain or partly immersed in water). Observed data are represented by circles proportional to the number of samples collected and coloured according to random-effect coefficients for sampling locations. Details of the binomial generalized linear mixed model are given in table 3.
(TIFF)

S1 Appendix Effects of storage time and faecal quality on elephant DNA concentration. (A) Mean elephant DNA concentration (± SD) and (B) predicted elephant DNA concentration per week of storage for four categories of faecal quality. (C) Summary of the best truncated negative binomial generalized linear mixed model using 396 faecal DNA extracts. Faecal quality was categorized into four groups: very fresh, fresh (reference category), reduced surface and degraded. Sampling location was included as random effect.
(DOCX)
