# Supplementary material for: Improving cost-efficiency of faecal genotyping: New tools for elephant species
Source: PLoS One. 2019 Jan 30;14(1):e0210811. doi: 10.1371/journal.pone.0210811 (PMC6353156; doi:10.1371/journal.pone.0210811)
Supplement: S1 Table — Faeces were scrubbed using a buccal swab (Isohelix, Cell projects) and preserved into storage buffer (500 μl of LS buffer and 25 μl of proteinase K, Stabilizing kit, Isohelix, Cell Projects). The protocol is derived from the QIAamp Fast Stool Mini kit (51604) protocol (QIAGEN) and the Isohelix DNA Isolation kit (DDK-50) protocol (Cell Project). (DOCX) [file pone.0210811.s001.docx]

# Supporting Information

Bourgeois S., J. Kaden, H. Senn, N. Bunnefeld, K. J. Jeffery, E. F. Akomo-Okoue, R. Ogden, and R. McEwing. 2018. Improving Cost-efficiency of Faecal Genotyping: new tools for Elephant Species.

Table S1. A modified protocol for DNA extraction from faecal samples collected using a buccal swab (Isohelix, Cell projects) and preserved into storage buffer (500 µl of LS buffer and 25 µl of proteinase K, Stabilizing kit, Isohelix, Cell Projects). The protocol is derived from the QIAamp Fast Stool Mini kit (51604) protocol (QIAGEN) and the Isohelix DNA Isolation kit (DDK-50) protocol (Cell Project).

| Step | Description |
| --- | --- |
| 1 | Vortex the 2-ml tube containing the sample (swab tip in buffer solution). Centrifuge during 2 min (14,100 g). Discard the swab. |
| 2 | Pipette supernatant into a clean 1.5ml tube (~ 450ul). Add 250 µl InhibitEx buffer and vortex immediately for at least one minute. Leave for a minute at ambient temperature. |
| 3 | Centrifuge for 2 min (14,100 g). Pipette supernatant into a new 1.5 ml tube containing 25 µl of proteinase K. Vortex. |
| 4 | Incubate at 56°C for 1 hour. |
| 5 | Centrifuge for 2 min (14,100 g). If stool particles deposited at bottom of tube, pipette supernatant into a clean 1.5 ml tube. |
| 6 | Add 500 µl CT solution and vortex immediately. |
| 7 | Pipette 600 µl into the QIAamp spin column. Centrifuge 1 min (14,100 g). Place the pin column into a new collection tube and repeat until all the lysate has been loaded on the spin column. Place the spin column into a new collection tube. |
| 8 | Add 500 µl buffer AW1. Centrifuge 1 min (14,100 g). Place the spin column into a new collection tube. |
| 9 | Add 500 µl buffer AW2. Centrifuge 1 min (14,100 g). Place the spin column into a new collection tube. |
| 10 | Centrifuge 3 min (14,100 g). Place the spin column into a clean 1.5 ml tube. |
| 11 | Add 75 µl buffer ATE. Incubate 2 min at 56°C. Centrifuge 1 min (14,100 g). |
