# Supplementary figures and images for: Improving cost-efficiency of faecal genotyping: New tools for elephant species
Source: PLoS One. 2019 Jan 30;14(1):e0210811. doi: 10.1371/journal.pone.0210811 (PMC6353156; doi:10.1371/journal.pone.0210811)

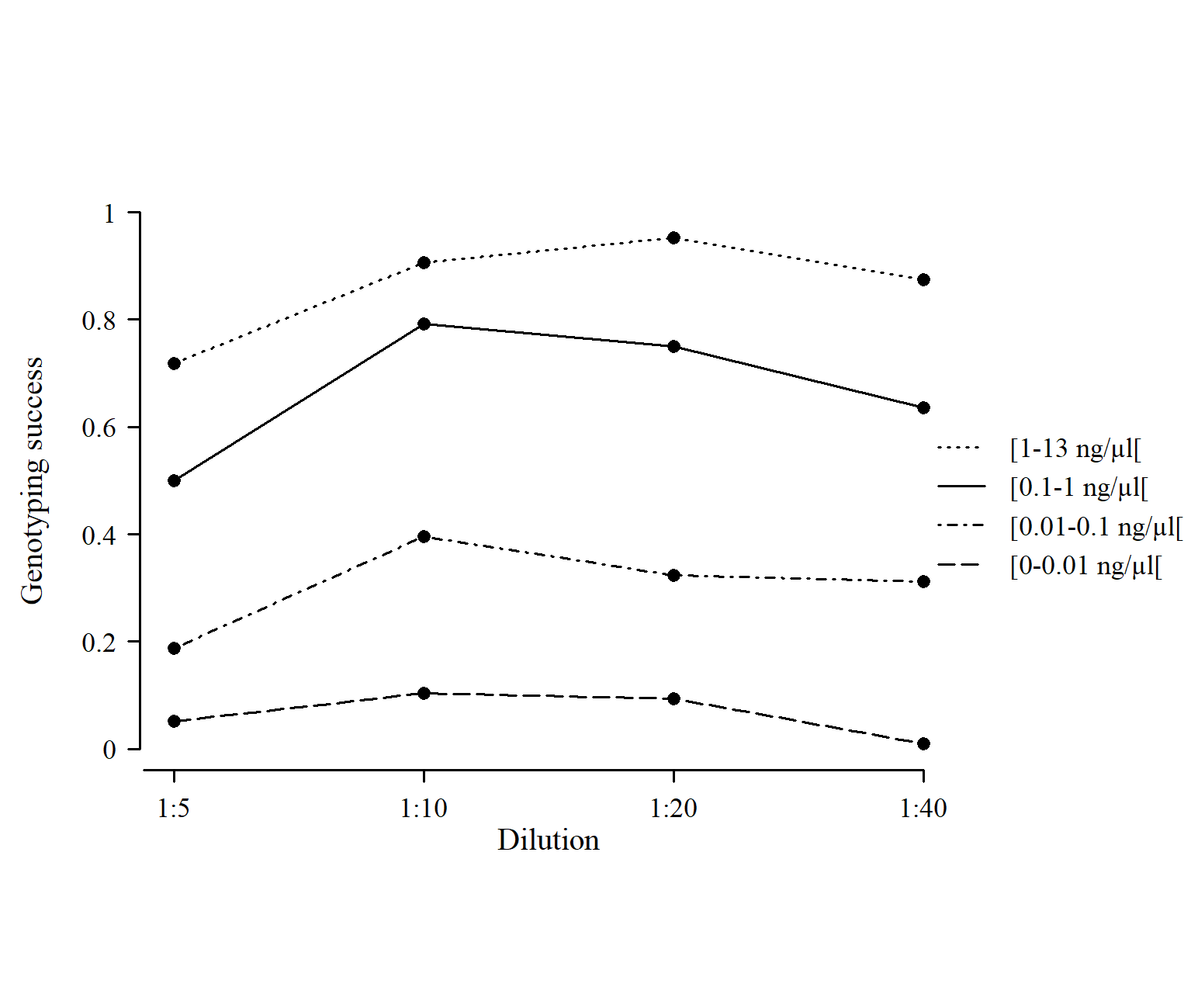

Supplement: S1 Fig — The pilot study included 88 faecal DNA extracts DNA extracts classified into four categories based on target DNA concentration. (TIFF) [file pone.0210811.s003.tiff]

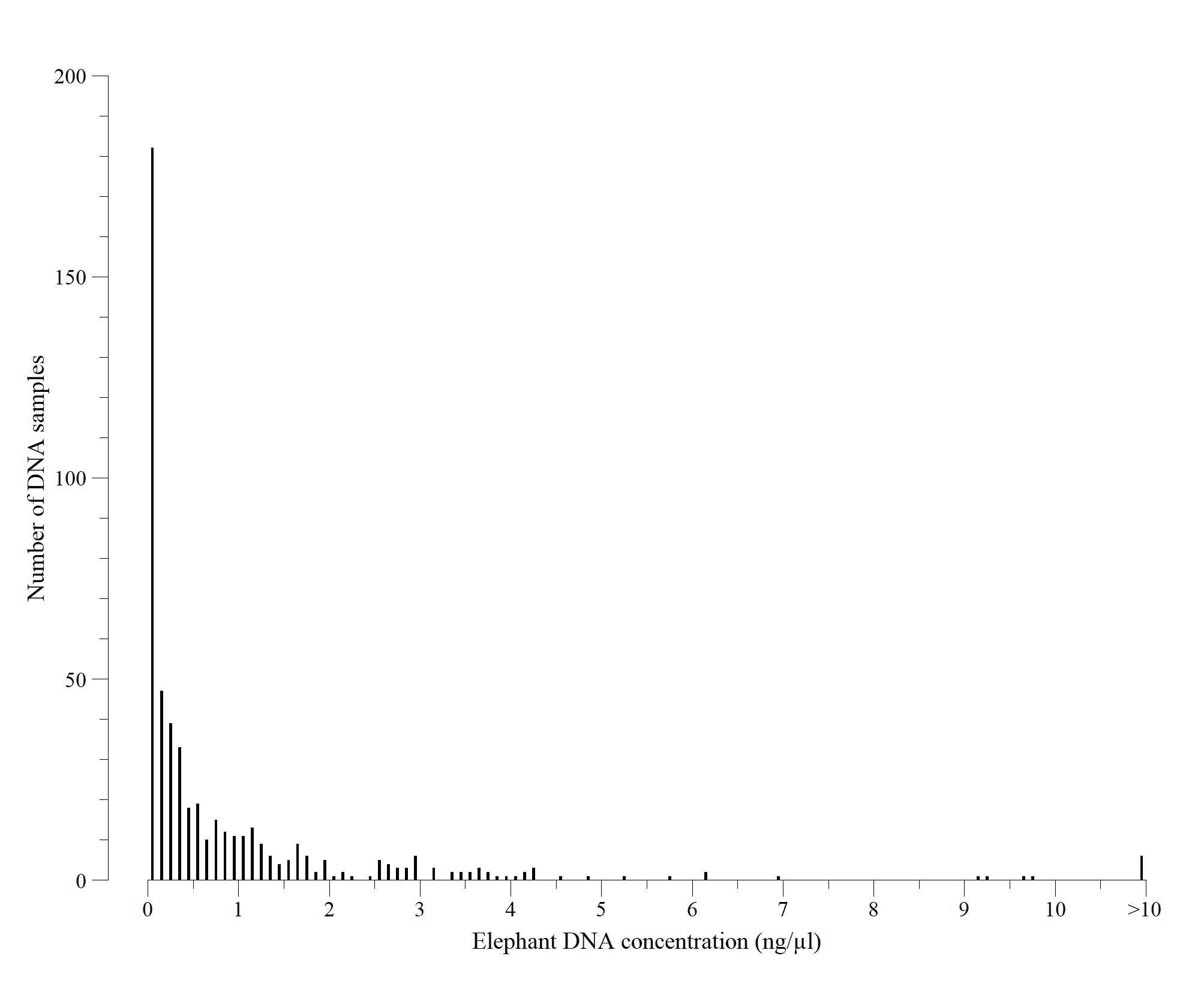

Supplement: S2 Fig — DNA concentrations ranged between 0 and 28.0 ng/μl. Samples were preserved into a lysis buffer and elution volume was 75 μl for all samples. (TIFF) [file pone.0210811.s004.tiff]

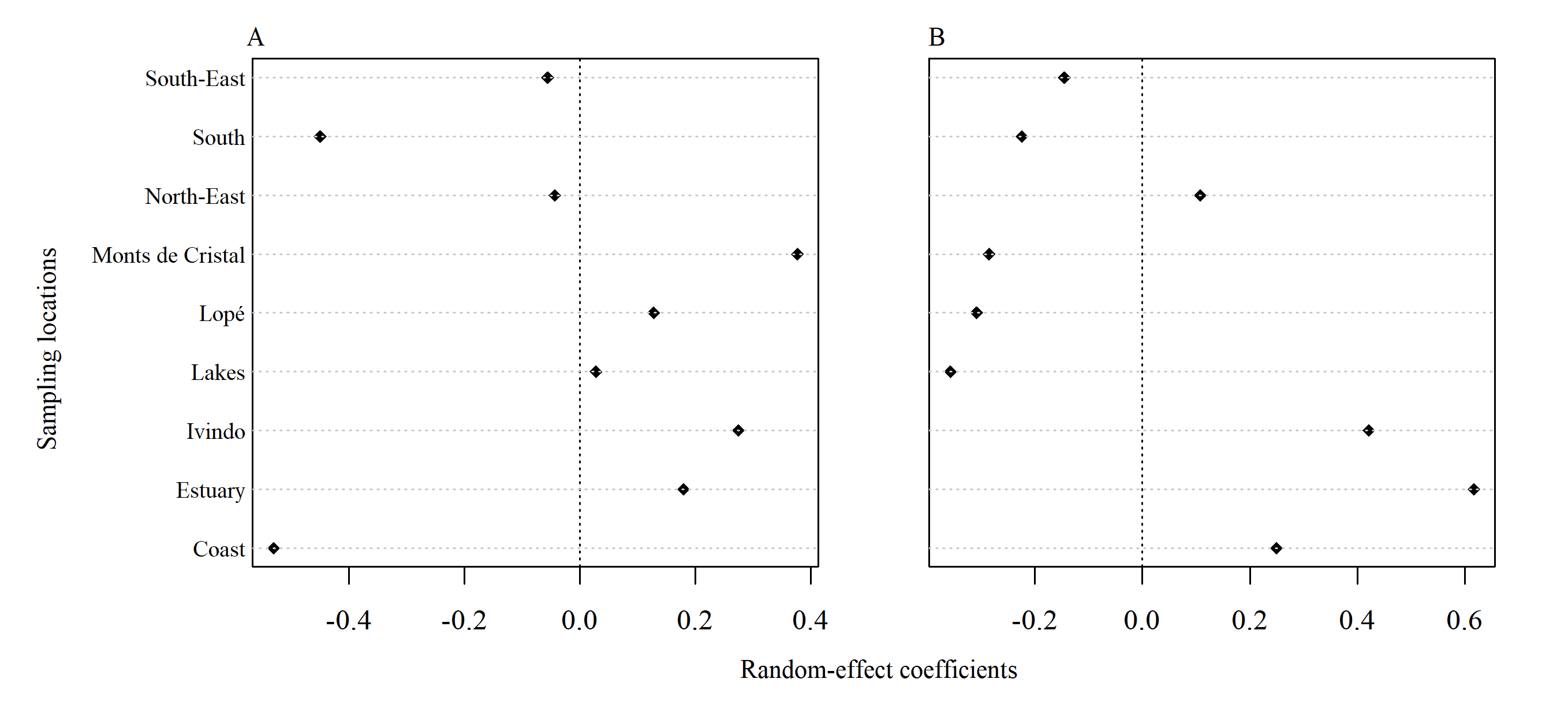

Supplement: S3 Fig — The model included (A) the best binomial generalized linear mixed model for the effects of storage time and faeces quality on elephant DNA extraction success, and (B) the best truncated negative binomial generalized linear mixed model for the effects of storage time and faeces quality on elephant DNA concentration. (TIFF) [file pone.0210811.s005.tiff]

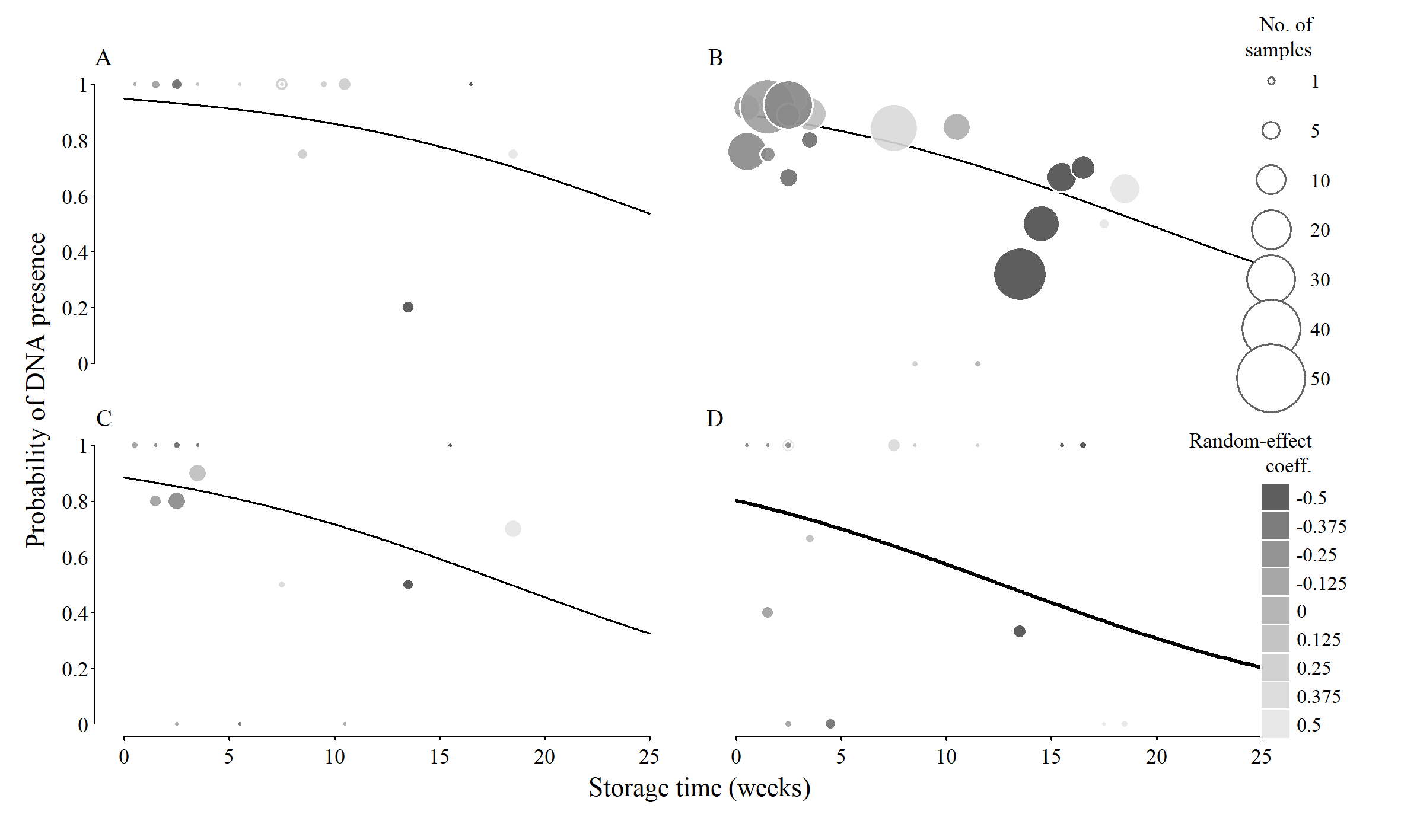

Supplement: S4 Fig — The four categories of faecal quality were: (A) very fresh (collected within 1 hour after defecation), (B) fresh (collected within 24 hours after defecation), (C) reduced surface (less than 24 hours old but partly destroyed by insects or exposed to direct sunlight), and (D) degraded (collected between 24 and 48 hours after defecation or found after rain or partly immersed in water). Observed data are represented by circles proportional to the number of samples collected and coloured according to random-effect coefficients for sampling locations. Details of the binomial generalized linear mixed model are given in table 3. (TIFF) [file pone.0210811.s006.tiff]
