# Supplementary material for: Improving cost-efficiency of faecal genotyping: New tools for elephant species
Source: PLoS One. 2019 Jan 30;14(1):e0210811. doi: 10.1371/journal.pone.0210811 (PMC6353156; doi:10.1371/journal.pone.0210811)
Supplement: S1 Appendix — (A) Mean elephant DNA concentration (± SD) and (B) predicted elephant DNA concentration per week of storage for four categories of faecal quality. (C) Summary of the best truncated negative binomial generalized linear mixed model using 396 faecal DNA extracts. Faecal quality was categorized into four groups: very fresh, fresh (reference category), reduced surface and degraded. Sampling location was included as random effect. (DOCX) [file pone.0210811.s007.docx]

Supporting Information

Improving Cost-efficiency of Faecal Genotyping: new tools for Elephant Species.

Bourgeois S., J. Kaden, H. Senn, N. Bunnefeld, K. J. Jeffery, E. F. Akomo-Okoue, R. Ogden, and R. McEwing. 2018. Plos One.


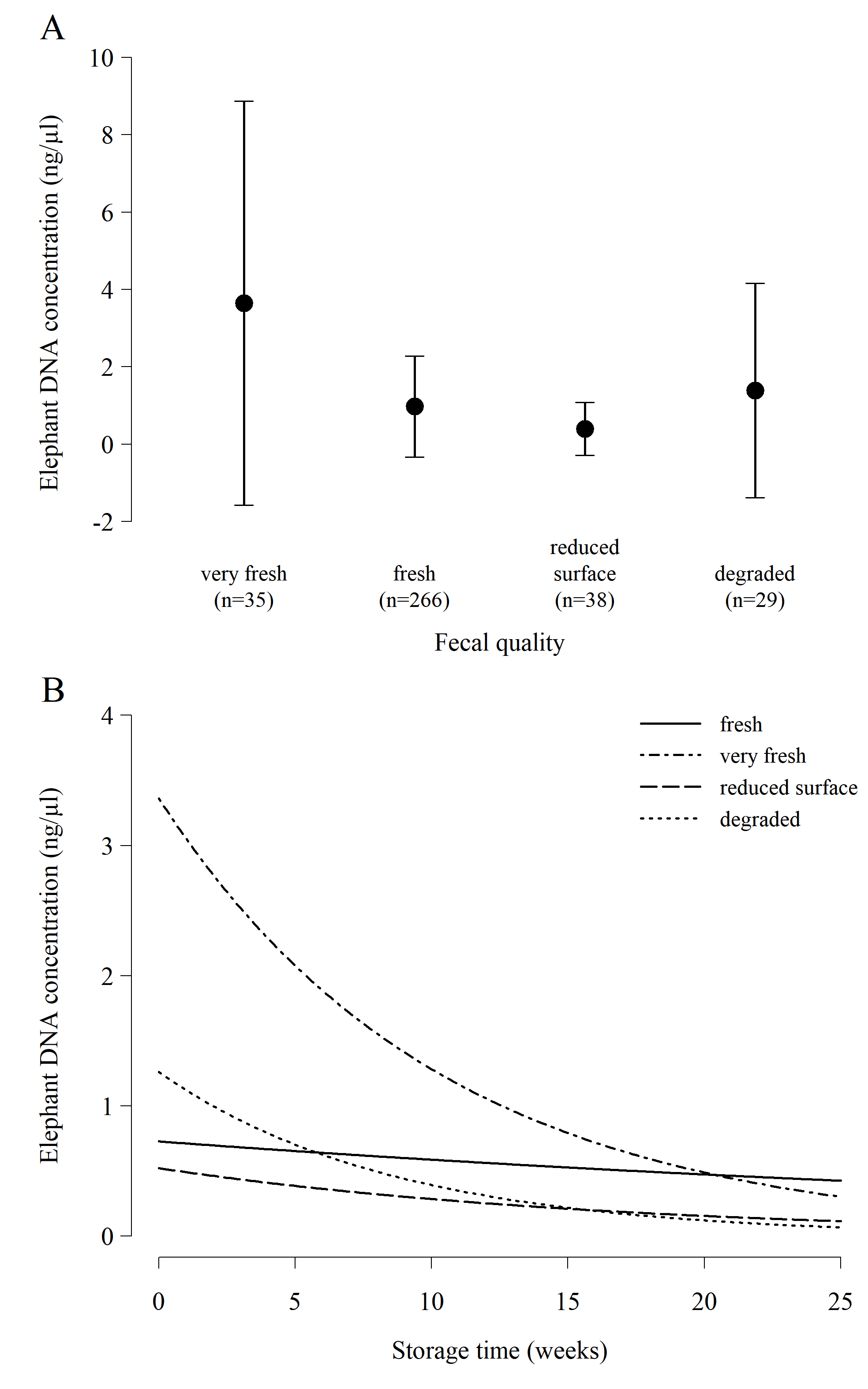


C

| Variable | Coeff. (±SE) | Z | p-value |
| --- | --- | --- | --- |
| Fixed effects |  |  |  |
| Intercept | 6.428 ±0.131 | 49.23 | <0.001 |
| Storage time | -0.126 ±0.079 | -1.59 | 0.112 |
| Very fresh | 0.959 ±0.123 | 7.80 | <0.001 |
| Reduced surface | -0.627 ±0.153 | -4.11 | <0.001 |
| Degraded | -0.176 ±0.160 | -1.10 | 0.272 |
| Storage time:very fresh | -0.442 ±0.118 | -3.74 | <0.001 |
| Storage time:reduced surface | -0.230 ±0.147 | -1.57 | 0.117 |
| Storage time:degraded | -0.562 ±0.200 | -2.81 | 0.005 |
| Random effects |  |  |  |
| No. groups | 9 |  |  |
| Variance | 0.126 |  |  |
| SD | 0.355 |  |  |

S1 appendix. Effects of storage time and faecal quality on elephant DNA concentration. A) Mean elephant DNA concentration (± SD) and B) Predicted elephant DNA concentration by week of storage for four categories of feacal quality. C) Summary of the best truncated negative binomial generalized linear mixed model using 396 faecal DNA extracts. Faecal quality was categorized into four groups: very fresh, fresh (reference category), reduced surface and degraded. Sampling location was included as random effect.
